# CT and Sonographic Findings of a Calcified Fibrin Sheath From an Umbilical Venous Catheter in a Neonate

**DOI:** 10.7759/cureus.21865

**Published:** 2022-02-03

**Authors:** Brandon G Brockbank, Paul Clark

**Affiliations:** 1 Radiology, Brooke Army Medical Center, San Antonio, USA

**Keywords:** central venous catheter complications, umbilical venous catheter, retained fibrin sheath, pediatrics, neonate, fibrin sheath

## Abstract

Central venous catheters are ubiquitous in current medical practice in intensive care units and for long-term nutrition, chemotherapy, and antibiotic therapies. Umbilical venous catheters provide short-term central vascular access and are used in the neonatal period. This case presents sonographic and CT imaging findings of an intrahepatic and intracardiac calcified fibrin sheath following removal of a short-term umbilical venous catheter in a 32-day-old premature neonate.

## Introduction

Umbilical venous catheters are a mainstay of advanced medical care in the neonatal intensive care unit (NICU), where neonates may require central venous access for total parenteral nutrition, antibiotics, hemodialysis, and other interventions. There are well-documented complications in the literature from central venous catheters (CVC), and specifically for umbilical venous catheters (UVC), including central line-associated bloodstream infections (CLABSIs), device failure, thrombosis, occlusion, migration, extravasation, and phlebitis [1]. Occlusion is one of the least reported complications in UVCs [2]. It typically results from the formation of a fibrin sheath, adherence of the UVC tip to the vessel wall, or thrombosis of the UVC tip [3]. This case is the first published case in English of sonographic and CT findings of a calcified fibrin sheath in a neonate secondary to an umbilical venous catheter.

## Case presentation

**Figure 1:**
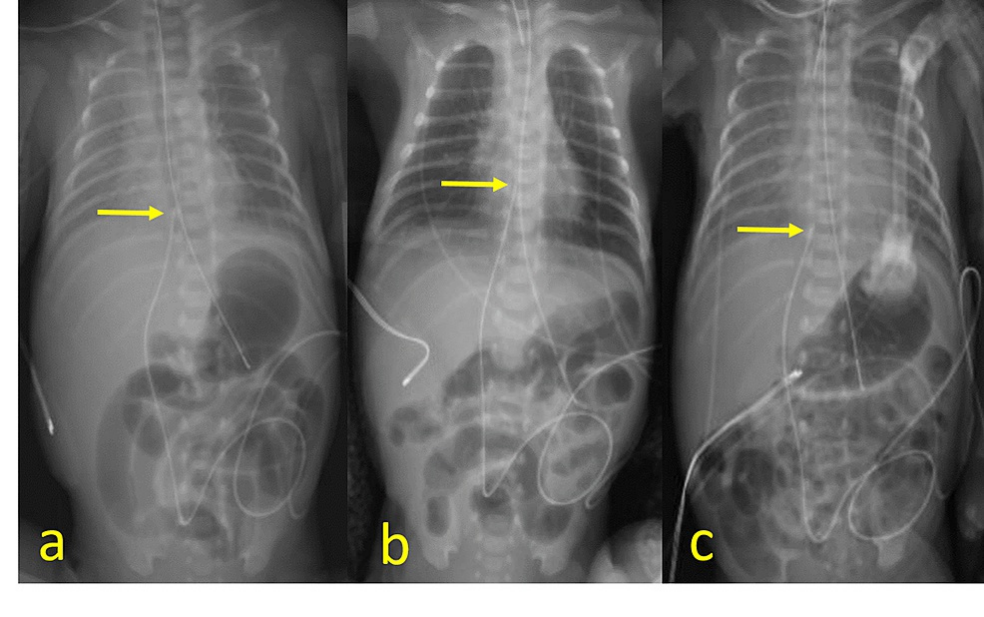
Supine portable radiographs on day 1 (a), day 2 (b), and day 5 (c) demonstrate a UVC with tip near the inferior cavoatrial junction (arrow, day 1), in the right atrium (arrow, day 2), and again near the inferior cavoatrial junction (arrow, day 5). On Day 8, a PICC was placed and the UVC was removed UVC: umbilical venous catheter; PICC: peripherally inserted central catheter

A preterm infant delivered at Gestational Age (GA) of 31+1 weeks via caesarian section due to maternal preeclampsia required neonatal intensive care for respiratory distress. A 5Fr double-lumen UVC was placed on day 1 of life with the distal tip positioned at the inferior cavoatrial junction. Follow-up daily radiographs were obtained to monitor the position of the invasive devices (endotracheal tube, UVC, enteric tube, etc.) and the cardiorespiratory condition. These radiographs demonstrate cranial migration of the catheter with the tip in the right atrium on day 2. On day 5, the catheter tip was repositioned near the inferior cavoatrial junction (Figure 1).

**Figure 2:**
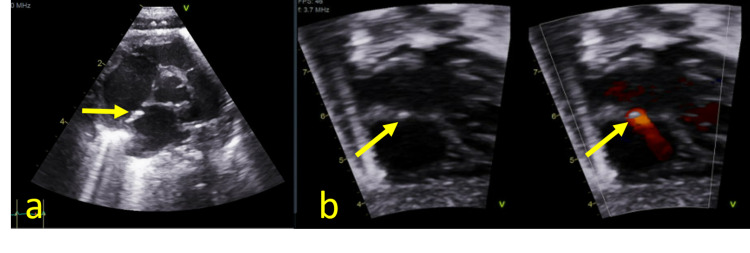
Transthoracic echocardiogram on day 32 with 5-chamber (a) and 4-chamber (b) views with grayscale and color Doppler demonstrating a hyperechoic focus along the interatrial septum (arrows)

On day 8, a peripherally inserted central catheter (PICC) was placed for continued long-term total parenteral nutrition (TPN), and the umbilical venous catheter was removed. The patient continued to require neonatal intensive care and had a complicated course requiring multiple intubations. On day 32, a transthoracic echocardiogram was obtained to evaluate pulmonary hypertension as a potential cause of the patient’s ongoing respiratory difficulties. This exam demonstrated a hyperechoic focus along the interatrial septum which represented a fibrin sheath (Figure 2).

The neonate continued to have respiratory distress and on day 48, a chest CT was obtained to evaluate for interstitial lung disease (igure 3). This exam demonstrated a curvilinear hyperdensity with attenuation of 333 Hounsfield units extending from the intrahepatic inferior vena cava (IVC) to the right atrium.

**Figure 3:**
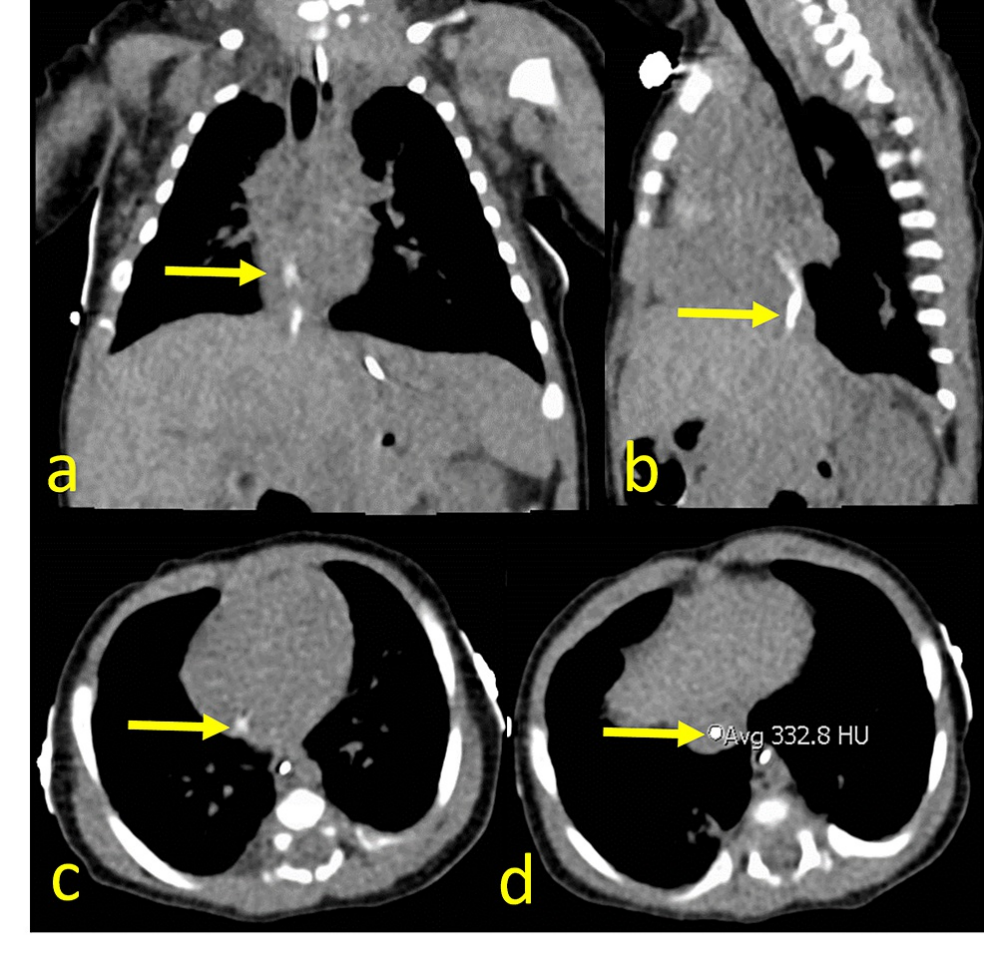
Coronal (a), sagittal (b), and axial (c, d) CT images on day 48 demonstrate a non-occlusive curvilinear hyperdensity extending from the intrahepatic inferior vena cava to the right atrium (arrows). Incidental note of enteric tube in the esophagus and stomach

**Figure 4:**
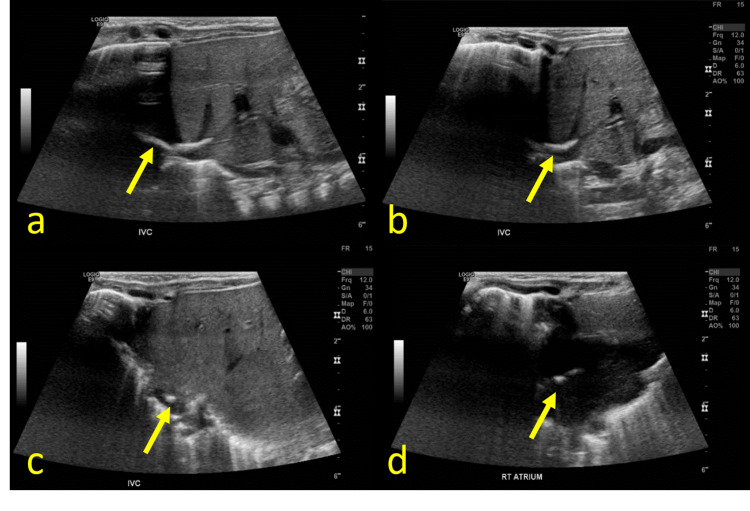
Grayscale transverse (a, b) and axial (c, d) ultrasound images of the right upper quadrant demonstrates a non-occlusive curvilinear hyperechoic structure extending from the confluence of the left hepatic vein and ductus venosus through the intrahepatic IVC to the right atrium (arrows) IVC: inferior vena cava

An ultrasound was performed and corroborated this finding. It demonstrated a curvilinear hyperechoic structure without significant posterior shadowing extending from the central left hepatic vein through the IVC to the right atrium (Figure 4).

**Figure 5:**
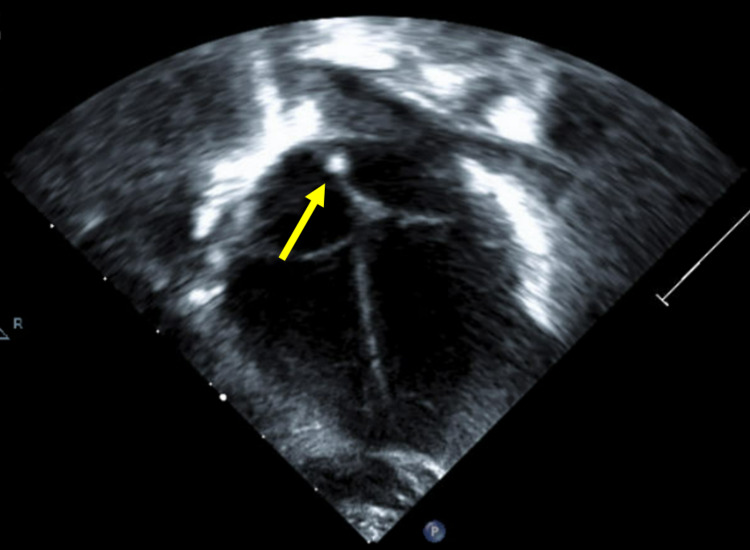
Transthoracic echocardiogram on day 127 with grayscale 4-chamber view demonstrating echogenic foci along the posterior interatrial septum (arrows). No residual curvilinear hyperechoic structure in the hepatic IVC or right atrium IVC: inferior vena cava

The patient remained in the neonatal intensive care unit (NICU) and obtained a follow-up echocardiogram on day 61, which demonstrated similar findings to prior. Anticoagulation therapy was deferred because the patient was scheduled to undergo a lung biopsy. The patient’s respiratory status improved and the patient did not undergo a biopsy and was ultimately discharged home after more than 3 months in the NICU. A follow-up outpatient echocardiogram was performed on day 127 of life, demonstrating a hyperechoic focus along the interatrial septum. There was no residual calcified fibrin sheath in the right atrium or IVC (Figure 5). No further imaging follow-up was obtained.

## Discussion

Umbilical venous catheters provide short-term central venous access in the neonatal period. If long-term vascular access is required, a PICC is preferred [4]. Two recent systematic reviews found that the rate of UVC occlusion is 0.2-2.3%, which is likely partially because UVCs are not intended for long-term use and are typically removed prior to clinically apparent occlusion [1-2]. Catheter occlusion manifests clinically as the inability to aspirate from a catheter that flushes easily and is typically the result of a fibrin sheath, a catheter tip thrombus, or the catheter becoming adherent to the vessel wall [3]. UVCs are ideally positioned in the supradiaphragmatic inferior vena cava, but commonly migrate as the neonate grows and the umbilical stump dries [2]. This patient initially had appropriate positioning, but the tip was in the right atrium for three days before repositioning to the inferior cavoatrial junction due to catheter migration.

Catheter-associated fibrin sheath formation has been described in swine models with the development of a cellular covering along the catheter from activation of the coagulation cascade. This covering is made of smooth muscle cells, thrombus, endothelial cells, and plasma proteins like fibrinogen after the first week. As time progresses, pathophysiological changes like smooth muscle and endothelial cell proliferation occur and ultimately the formation of collagen and fibrous connective tissue [5].

CVC-associated fibrin sheaths are very common in the adult population, with one study of 133 hemodialysis patients reporting the formation of fibrin sheaths in 47% at the time of removal [6]. In pediatric patients with PICCs, one study found four of 258 (1.6%) patients developed fibrin sheaths which were discovered at the time of catheter removal [7]. Retained fibrin sheaths after CVC removal were found on CT in 14% of adults in a retrospective study [8]. Multiple case reports in adults describe imaging and treatment of retained fibrin sheaths [9]. A few pediatric case reports and case series have been published about retained fibrin sheaths, with the median age of 8 years (ages 0-19 years) and median catheter dwell time of 2 years (6 days to 4 years) (Table 1). In neonates specifically, there are two published cases of infants with retained fibrin sheaths; one was a term infant with a PICC that was present for 49 days [10] and the other a term infant with a UVC present for 6 days [11].

**Table 1:** Pediatric cases of retained fibrin sheaths and their outcomes

Age (years)	Diagnosis	Catheter dwell time	Outcome	Study
Neonate	Meconium Aspiration Syndrome	49 days	Surgery	Anderson [10]
Neonate	Pulmonary Hypertension	6 days	Anticoagulation	Ríos-Méndez [11]
4	Acute Lymphoblastic Leukemia	2.5 years	Observation	Hughes [12]
6	Acute Lymphoblastic Leukemia	1.5 years	Cardiac Surgery	Fabi [13]
6	Acute Lymphoblastic Leukemia	1.5 years	Observation	Keehn 2015 [14]
7	Acute Lymphoblastic Leukemia	2 years	Cardiac Surgery	Massardier [15]
9	Non-Hodgkin Lymphoma	4 years	Cardiac Surgery	Massardier [15]
11	Chronic Heart Failure	10 days	Surgery	Mogi [16]
11	Juvenile Dermatomyositis	Not reported	Cardiac Surgery	Kira [17]
12	Acute Lymphoblastic Leukemia	0.5 years	Pulmonary Embolism, observation	Rousslang [18]
15	Acute Lymphoblastic Leukemia	3.5 years	Observation	Sabbaghian [19]
17	Acute Lymphoblastic Leukemia (Li-Fraumeni Syndrome)	2 years	Cardiac Surgery	Massardier [15]
19	Acute Lymphoblastic Leukemia	3.5 years	Anticoagulation	Van Bastelaar [20]

In the previously reported cases, the clinical severity of a retained fibrin sheath varies. Some were asymptomatic and observed over time, others were asymptomatic and treated with anticoagulation, one resulted in an asymptomatic pulmonary embolism, and multiple required cardiac surgery for removal of fibrin sheaths (Table 1). This patient ultimately did not require treatment and remained asymptomatic from this non-occlusive retained fibrin sheath.

## Conclusions

Retained fibrin sheaths are relatively rare complications of central venous catheters and are typically involved in long-term catheters. These sheaths can have an innocuous clinical course, but in some cases may require invasive surgery for removal. This is the first case report in English literature describing a retained fibrin sheath in a neonate following removal of a UVC with both CT and sonographic findings. This case highlights the fact that fibrin sheaths can form after short-term use (8 days in this case) in neonates and provides additional support for the earliest possible removal of central venous catheters, including umbilical venous catheters.
